# Gastric emptying scintigraphy: Comparison of imaging times for diagnosing rapid gastric emptying, a single center experience

**DOI:** 10.1097/MD.0000000000042246

**Published:** 2025-04-25

**Authors:** Cibele Luna, Laura Palacio, Sam Samaan

**Affiliations:** a Department of Radiology, Division of Abdominal Imaging, University of Miami, Jackson Memorial Hospital, Miami, FL; b Department of Radiology, Division of Nuclear Medicine, University of Miami, Jackson Memorial Hospital, Miami, FL; c Department of Radiology, Division of Nuclear Medicine, Baylor College of Medicine, Houston, TX.

**Keywords:** gastric emptying scintigraphy, nuclear medicine, rapid gastric emptying

## Abstract

This study aimed to evaluate the diagnostic utility of the 30- and 60-minute imaging time points for rapid gastric emptying (RGE) using gastric emptying scintigraphy. The primary objective was to determine whether the 30-minute time point, unique to the Miami Method, provides additional diagnostic value compared to the standard 60-minute time point recommended by the National Standard Protocol. A retrospective analysis was conducted on consecutive patients who underwent gastric emptying scintigraphy between 2015 and 2021. Patients were included if they completed imaging at both 30 and 60 minutes using a hybrid protocol incorporating the Miami Method and the National Standard Protocol. The McNemar’s test was used to compare the frequency of RGE diagnosis at the 30- and 60-minute time points and Cohen’s Kappa coefficient to assess the inter-time point agreement. Among the 38 patients diagnosed with RGE, 24 patients (63%) were diagnosed with RGE only at 30 minutes, 6 patients (16%) were diagnosed with RGE only at 60 minutes, and 8 patients (21%) were diagnosed with RGE at both 30 and 60 minutes. The McNemar’s test showed a statistically significant difference (*P* = .0024) between the 30- and 60-minute time points, indicating that 30-minute imaging identifies a distinct group of patients who might not be diagnosed at 60 minutes. However, there was poor agreement between the 2 time points (Cohen’s Kappa = −0.34), suggesting that the 30-minute time point may need further validation. The 30-minute imaging time point detected more cases of RGE compared to the 60-minute time point, suggesting that it may capture early rapid emptying events missed at 60 minutes. However, the lack of agreement between the time points raises concerns about overdiagnosis. These findings highlight the need for further research to validate the clinical utility of the 30-minute time point and determine its impact on patient management and outcomes.

## 1. Introduction

Gastric emptying scintigraphy (GES) has been a critical tool for evaluating gastric motility disorders since its first use in 1966 with chromium-51-labeled food.^[1]^ Over time, it has evolved into a standardized method for quantifying gastric emptying, providing a physiologic, noninvasive, and quantitative assessment.^[2-4]^

Among the various applications of GES, its role in diagnosing rapid gastric emptying (RGE) or “dumping syndrome,” is of particular interest. RGE is well-recognized in postsurgical patients, but its pathophysiology remains unclear in those without prior gastric surgery.^[5]^ The widely accepted definition of RGE includes a retained meal value of <70% at 30 minutes or <30% at 1 hour.^[2,6]^ However, while these time points are integral to defining RGE, existing protocols incorporate multiple additional imaging time points, raising the question of whether all are necessary or if a more streamlined approach can yield equally reliable diagnostic results.

Current GES protocols vary in their approach to assessing RGE. The National Standard Protocol (NSP) recommends imaging at 0, 1, 2, and 4 hours,^[7,8]^ whereas the Miami Method, widely used at our institution, includes imaging at 0, 30, 60, 120, and 150 minutes, supplemented by delayed 4-hour imaging.^[9-11]^ However, no study has explicitly examined the comparative utility of the 30- and 60-minute time points in diagnosing RGE. This study aims to address this gap by evaluating the diagnostic performance of these specific time points using a standardized meal and a protocol that integrates both the Miami Method and the NSP.

## 2. Materials and methods

### 2.1. Study design and patient selection

This HIPAA-compliant IRB-approved (IRB #20220544) retrospective analysis analyzed consecutive patients who underwent GES in the Nuclear Medicine Department of Jackson Memorial Hospital between January 2015 and December 2021. The study was designed to specifically assess the diagnostic utility of the 30- and 60-minute time points for detecting RGE. Informed consent was waived by the IRB.

Patient data were retrieved using the institutional search tool within the Picture Archiving and Communication System (PACS). To minimize sampling bias, random selection of consecutive patients meeting inclusion criteria was performed, ensuring a representative sample of those undergoing GES within the study period. Additionally, exclusion criteria were applied systematically to reduce potential confounding factors that could affect gastric emptying, such as underlying metabolic conditions, prior gastric surgery, or protocol deviations. Exclusion criteria included:

- Ingested <50% of the standard meal.
- Received a non-standardized meal or liquid-only protocol.
- Had blood glucose >200 mg/dL at the time of imaging.
- Had suspected gastric outlet obstruction.
- Took >10 minutes to ingest the meal.
- Had prior gastric surgery.
- Had an aborted scan.

By ensuring strict protocol adherence and excluding cases with confounding factors, the study aimed to enhance internal validity and improve the generalizability of its findings.

### 2.2. Gastric scintigraphy protocol

Patients were administered the standard solid-phase meal, consisting of 2 egg whites radiolabeled with Technetium-99m (Tc-99m) sulfur colloid, 2 slices of bread, 30 g of strawberry jam, and 120 mL of water.

**Figure 1.**
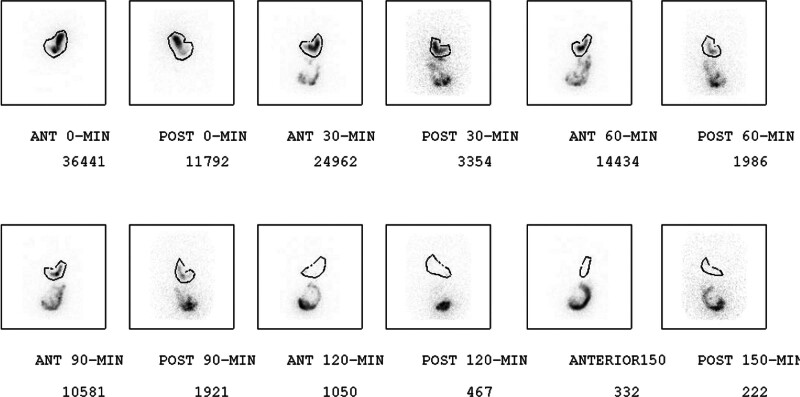
Sequential images for Gastric Emptying Protocol. GES demonstrates correct regions of interest in anterior and posterior projections. Anterior and posterior projections are performed every 30 min for the first 2 and 1/2 h and at 4 h post-meal ingestion (not shown). GES = gastric emptying scintigraphy.

After meal ingestion, imaging was performed in upright anterior and posterior projections according to our institutional protocol (Fig. 1). The study was specifically designed to evaluate the 30- and 60-minute time points, as these are the defining thresholds for RGE.

**Figure 2.**
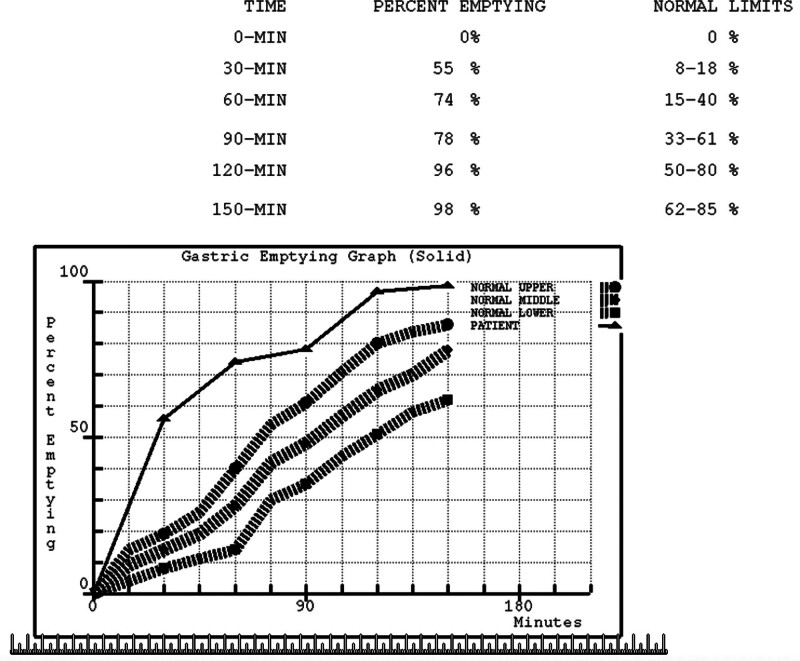
Miami Method curve. The Miami Method consists of a standard graph from the average population of Miami describing the emptied percentage of the radiolabeled meal at 0, 30, 60, 90, 120, and 150 min. The patient demonstrates RGE as evidenced by an increased emptied percentage of radiolabeled meal within 2 to 3 standard deviations above the normal Miami mean curve. RGE = rapid gastric emptying.

**Figure 3.**
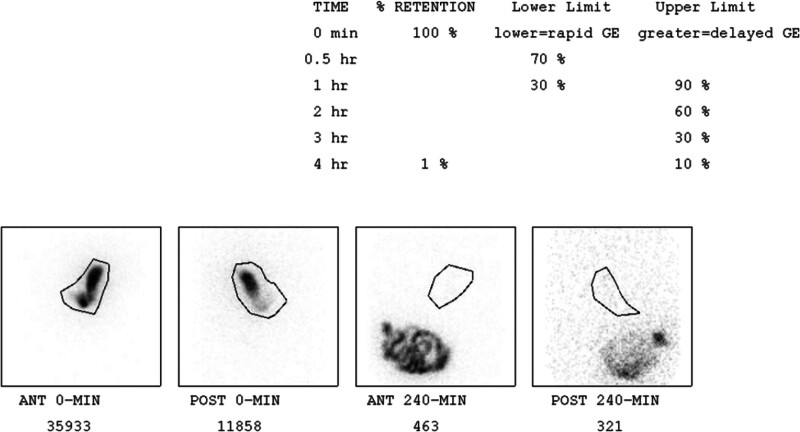
National Standard Protocol (NSP). The NSP describes the retained percentage at 0, 1, 2, and 4 h. NSP = National Standard Protocol.

All patients underwent imaging using a hybrid protocol, incorporating elements from both the Miami Method and the NSP:

- The Miami Method includes imaging at 0, 30, 60, 90, 120, and 150 minutes (Fig. 2). However, for this study, only the 30- and 60-minute time points were analyzed, as they are most relevant for diagnosing RGE.
- The NSP protocol includes imaging at 0, 1, 2, and 4 hours (Fig. 3). These extended time points are primarily used for assessing delayed gastric emptying, which was not the focus of this study. Therefore, only the 1-hour (60-minute) time point from the NSP was utilized.

The 30-minute time point is unique to the Miami Method, and this study aimed to determine whether its inclusion improves diagnostic accuracy. All patients included in this study were assessed with both protocols, allowing direct comparison of the 30- and 60-minute time points.

Gastric emptying quantification was performed using geometric mean values of anterior and posterior views.

### 2.3. Diagnostic criteria for rapid gastric emptying

RGE was defined using the widely accepted thresholds:

- Retained meal percentage <70% at 30 minutes.
- Retained meal percentage <30% at 60 minutes.

Patients were classified into 3 diagnostic categories:

1. RGE detected only at 30 minutes.
2. RGE detected only at 60 minutes.
3. RGE detected at both 30 and 60 minutes.

### 2.4. Data retrieval

GES images and reports were reviewed by a Nuclear Medicine fellow and a Nuclear Medicine Physician. Data were extracted from PACS and recorded according to the 30- and 60-minute retention values.

To reduce potential bias, the reviewers were blinded to the patients’ clinical histories and outcomes during data collection and analysis. Additionally, standardized criteria were applied to ensure objective interpretation of GES results across all cases.

The datasets generated and analyzed during this study are not publicly available, as they were obtained from the institutional PACS. However, they are available from the corresponding author upon reasonable request.

### 2.5. Statistical analysis

The McNemar’s test (paired *χ*² test) was used to compare the diagnosis of RGE at 30 minutes versus at 60 minutes to determine whether 30-minute imaging contributes unique diagnostic value or results in overdiagnosis. The inter-time point agreement analysis was conducted with Cohen’s Kappa coefficient. A *P*-value <.05 was considered statistically significant. Statistical analyses were performed using Python software version 3.8.

## 3. Results

### 3.1. Patient characteristics

**Figure 4.**
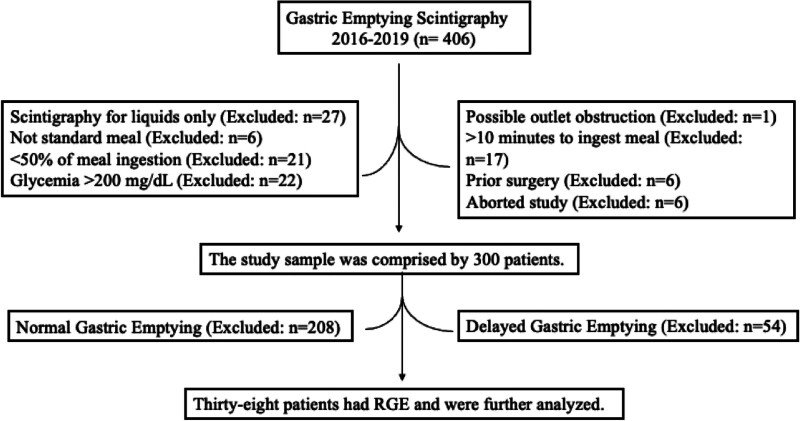
Study sample. Flowchart of patient enrollment, exclusion criteria, and final study sample.

Figure 4 demonstrates the flowchart of patient enrollment, exclusion criteria, and final study sample selection. A total of 406 patients underwent GES between 2016 and 2019. Patients were excluded for the following reasons: 27 patients had scintigraphy for liquids only, 6 cases did not use the standard meal, 21 patients were unable to ingest >50% of the meal, 22 patients had blood sugar >200 mg/dL, 1 patient had possible outlet obstruction, 17 patients consumed the radiolabeled meal in more than 10 minutes, 6 patients had prior surgery, and the study was aborted in 6 patients. The study sample was comprised of 300 patients. Of this cohort, 208 patients had normal gastric emptying, and 54 had delayed gastric emptying; therefore, they were excluded. Thirty-eight patients had RGE and were further analyzed. The median age was 64 years. Twenty-eight patients (74%) were males, and 10 (26%) were females.

### 3.2. Rapid gastric emptying quantification

**Figure 5.**
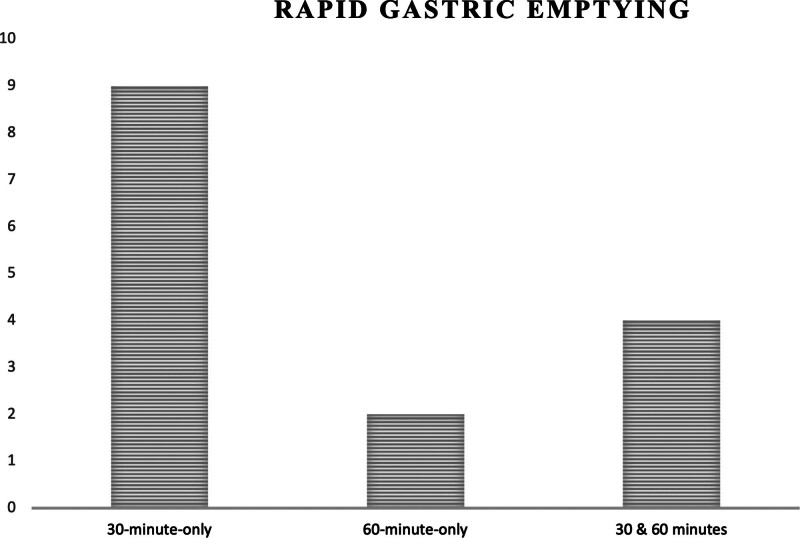
Rapid Gastric Emptying results at different imaging times. Twenty-four (63%) were diagnosed with RGE only at 30 min; 6 patients (16%) were diagnosed with RGE only at 60 min, and 8 patients (21%) were diagnosed with RGE at both 30 and 60 min. RGE = rapid gastric emptying.

GES results were analyzed at 30- and 60-minute time points to evaluate the frequency of RGE detection. Among the 38 patients diagnosed with RGE: Twenty-four (63%) were diagnosed with RGE at 30 minutes only compared to 6 patients (16%) diagnosed with RGE at 60 minutes only. Eight patients (21%) were diagnosed with RGE at both 30 and 60 minutes. These findings suggest that the 30-minute imaging time point detected a higher proportion of RGE cases compared to the 60-minute time point alone (Fig. 5).

### 3.3. Assessment of diagnostic performance of 30-minute imaging

The McNemar’s test yielded a statistically significant result (*χ*² = 9.20, *P* = .0024), indicating that there is a significant difference in RGE detection rates between the 2 time points.

The Cohen’s Kappa coefficient was −0.34, indicating poor agreement between the 30- and 60-minute time points beyond chance levels. This finding suggests that the 30-minute time point does not strongly correlate with the 60-minute diagnosis, highlighting the need for further investigation into whether it provides unique diagnostic value or contributes to overdiagnosis.

## 4. Discussion

GES is the standard procedure for evaluating gastric motility disorders due to its ability to provide a physiological, noninvasive, and quantitative assessment of gastric emptying. Multiple societies, including the Society of Nuclear Medicine (SNM), the American Gastroenterological Association (AGA), and the American Neurogastroenterology and Motility Society (ANMS), have issued guidelines for performing GES. Despite these guidelines, significant variability in GES protocols persists, particularly concerning the diagnostic criteria for RGE.^[12]^

The NSP for GES, recommended by most guidelines, includes imaging at 0, 1, 2, and 4 hours. Conversely, the Miami Method, which is utilized at our institution, incorporates a 30-minute time point. The decision to focus on the current study in the 30- and 60-minute time points was motivated by their direct relevance to RGE diagnosis, as RGE is typically defined by a retained meal value of <70% at 30 minutes or <30% at 1 hour.^[13]^

Our study demonstrated that the 30-minute imaging time point identified more cases of RGE compared to the 60-minute time point. Specifically, 63% of patients were diagnosed at 30 minutes only, whereas only 16% were diagnosed at 60 minutes only. The McNemar’s test revealed a statistically significant difference between the 30- and 60-minute imaging times (*P* = .0024). This finding suggests that the 30- and 60-minute imaging results are not interchangeable, and that the 30-minute time point identifies a distinct group of patients who might otherwise not be diagnosed at 60 minutes.

However, the poor agreement between the 2 time points (Cohen’s Kappa = −0.34) indicates that 30-minute imaging does not strongly correlate with the 60-minute diagnosis. This raises an important clinical consideration: whether the diagnostic criteria based on this time point are necessary or sufficient for accurately diagnosing RGE. This question is of particular importance because it could lead to significant changes in patient diagnosis and management.

Recent studies highlight significant variability in how RGE is defined and detected. The current study’s focus on a 30-minute time point is supported by prior work demonstrating that adding a 30-minute measurement can unmask RGE that would be missed by the 1-hour criterion alone. For example, Diaz et al found that including a 30-minute retention value diagnosed ~27% more RGE cases (those with <70% retention at 30 minutes but still normal at 1 hour) than using the 1-hour value alone.^[14]^

On the other hand, some studies have defined RGE using later time points or stricter thresholds – underscoring the lack of standardization. Lee et al identified RGE by <16% retention at 2 hours (based on the fastest quartile of normal values), and with this criterion found a 29% prevalence of RGE among patients tested for gastroparesis.^[15]^

Such different definitions can yield different patient classifications and prevalence rates. This diagnostic variability emphasizes the need for uniform criteria and has prompted calls for standardization in GES protocols for RGE. Notably, authors have pointed out that RGE is an under-recognized phenomenon in the literature, and inconsistent protocols historically have made it difficult to compare results across institutions.^[15]^ The current findings add to growing evidence that consistent inclusion of early time-point imaging (e.g., 30 minutes) improves detection of pathologically rapid emptying, potentially arguing for its routine incorporation into GES standards.

Despite these insights, no gold standard currently exists for defining optimal diagnostic criteria for RGE. The multi-institutional protocol by Tougas et al established normal gastric emptying values but did not define RGE-specific criteria.^[8]^ Previous studies, such as those by Abell et al, have evaluated models like area under the curve and total gastric emptying, but the diagnostic utility of the 30-minute time point remains largely unexplored.^[16]^ Our study contributes to filling this gap by demonstrating that earlier imaging may capture additional cases of RGE, yet also raises critical concerns about diagnostic consistency.

However, this study is not without limitations. It is a retrospective analysis with an inherent risk of selection bias, despite efforts to minimize this through consecutive patient sampling and strict exclusion criteria. Furthermore, the small sample size may have affected the statistical power of the analysis. The most critical implication of our findings is not simply whether the 30-minute time point should be routinely used, but rather that there is no universally accepted gold standard for RGE diagnosis. The observed discrepancy between the 30- and 60-minute results highlights a fundamental gap in the field – the lack of established, reliable diagnostic criteria for RGE. This uncertainty carries significant clinical implications, as it could alter the diagnosis for a substantial proportion of patients and impact management decisions.

Future studies must focus on establishing a consensus for RGE diagnostic criteria. Prospective, multi-institutional studies with larger sample sizes are needed to validate whether the 30-minute time point improves clinical outcomes or leads to unnecessary interventions. Furthermore, research should explore whether patient-specific factors, such as age, gender, diabetes status, and symptom severity, influence RGE diagnosis at different time points.

## 5. Conclusion

Our study provides novel insights into the diagnostic variability between the 30- and 60-minute imaging time points in GES. While the 30-minute time point identified additional cases of RGE, the lack of a gold standard raises concerns about its clinical relevance. The most pressing issue highlighted by our findings is the need to establish consistent and reliable diagnostic criteria for RGE, as the current lack of consensus may lead to significant differences in patient diagnosis and management.

## Acknowledgments

The authors acknowledge the use of ChatGPT for language editing, summarization, and idea refinement. All intellectual contributions, critical analysis, and final revisions were performed by the authors.

## Author contributions

**Conceptualization:** Sam Samaan.

**Data curation:** Cibele Luna, Sam Samaan.

**Formal analysis:** Cibele Luna, Sam Samaan.

**Investigation:** Cibele Luna, Laura Palacio, Sam Samaan.

**Methodology:** Cibele Luna, Sam Samaan.

**Supervision:** Sam Samaan.

**Writing – original draft:** Cibele Luna.

**Writing – review & editing:** Cibele Luna, Laura Palacio, Sam Samaan.
